# Multiple symmetric lipomatosis with chronic kidney disease and tophi

**DOI:** 10.1093/omcr/omad113

**Published:** 2023-10-23

**Authors:** Ruqi Tan, Pearl Pai

**Affiliations:** Department of Medicine, The University of Hong Kong-Shenzhen Hospital, Shenzhen, P.R. China; Clinical Medical Research Center, The Second Clinical Medical College of Jinan University, Shenzhen, P.R. China; Department of Medicine, The University of Hong Kong-Shenzhen Hospital, Shenzhen, P.R. China; Department of Medicine, University of Hong Kong Faculty of Medicine, Pokfulam, Hong Kong

## INTRODUCTION

Madelung’s disease (MD) is a rare condition characterized by benign and painless non-encapsulated symmetrical lipomatosis, affecting the neck, shoulders, upper arms, hips, and thighs and typically spares the face and distal limbs. The rate of progression can be fast in terms of months or slow in terms of years. Large fatty masses in the neck region may put pressure on and compromise function of surrounding structures causing dysphagia, dysphonia or difficulty in breathing. Diagnosis is made primarily through clinical history and physical examination supported by typical radiology imaging. The etiology of MD is unknown but the disease is often accompanied by numerous metabolic disorders. Here we described a 60 years old Chinese man with a history of chronic and heavy alcohol misuse. He had poorly controlled hypertension, gouty arthritis, hyperlipidemia and renal impairment and reported gradually enlarging masses in his neck region in the prior 6 months. Subsequent magnetic resonance imaging (MRI) revealed multiple large non-encapsulated lipomas in the neck and shoulder.

## CASE REPORT

A 60 years old Chinese man was admitted under nephrology because of poorly controlled hypertension and abnormal serum creatinine reported 3 weeks ago. He had hypertension for 10 years as well as gouty arthritis affecting predominantly the small joints of his hands. In addition, he had been noted for slowly growing fat masses in his neck and upper trunk in the past 6 months. He denied any difficulty breathing, swallowing or speaking, and the function of his arms were not affected. There was no significant family history. Some 6 months ago, he was commenced on perindopril, amlodipine, carvedilol, isosorbide mononitrate and febuxostat in one of the cardiology outpatient clinics. He had been a long-term smoker of 40 pack-years and an alcoholic consuming about 10 units of alcohol daily for 20 years until a few weeks before his hospital admission.

**Figure 1:**
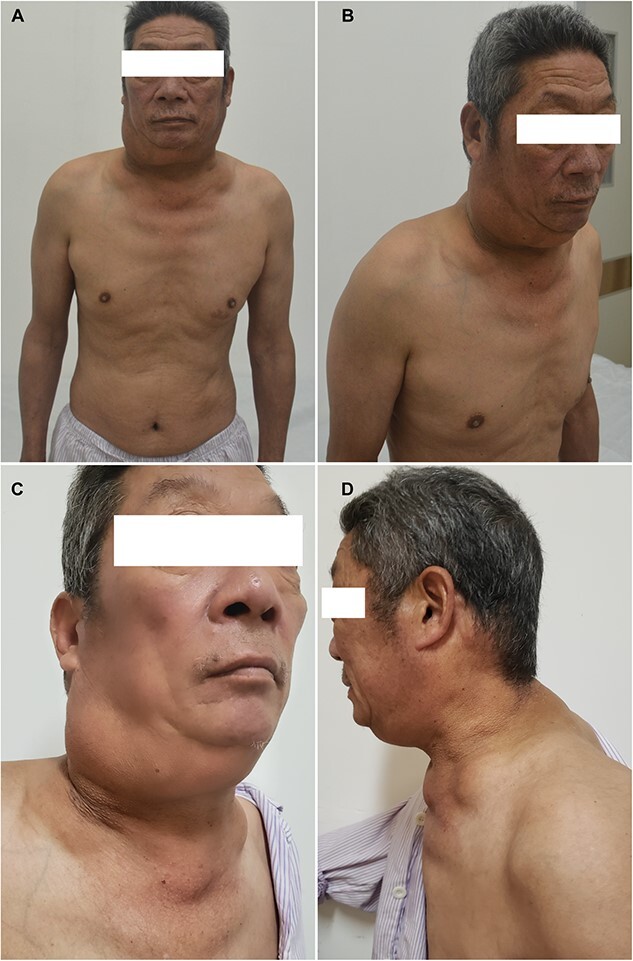
Symmetrical, soft, mobile masses around the neck, upper trunk, and bilateral upper arms.

**Figure 2:**
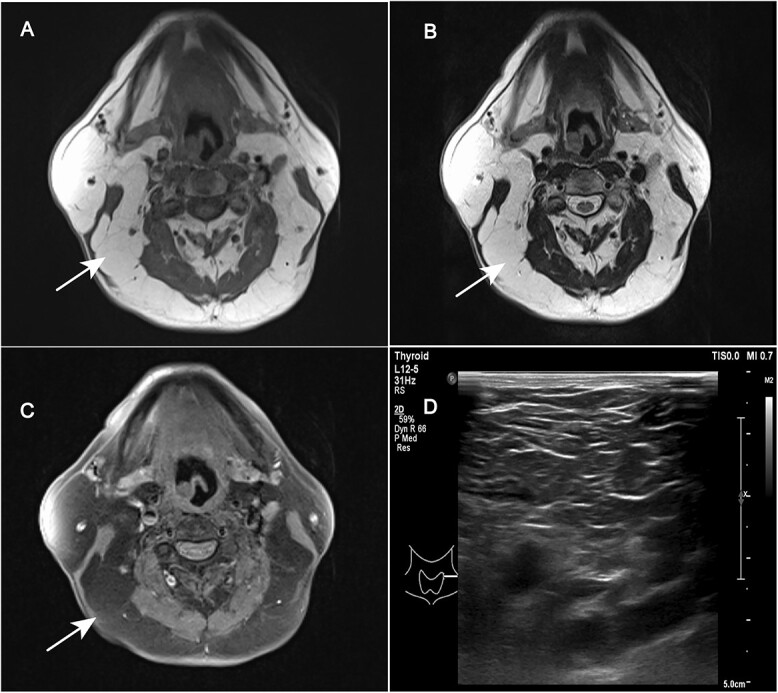
Magnetic resonance imaging of the head and neck: T1-weighted (**A**), T2-weighted (**B**) and fat suppressed imaging (**C**) showing non encapsulated soft tissue masses dispersed over the superficial and deep fascial spaces (Panel C, arrows). The color doppler ultrasound scan (**D**) of the masses showed irregular and diffuse thickening of subcutaneous fat tissue, without apparent capsules and borders.

**Figure 3:**
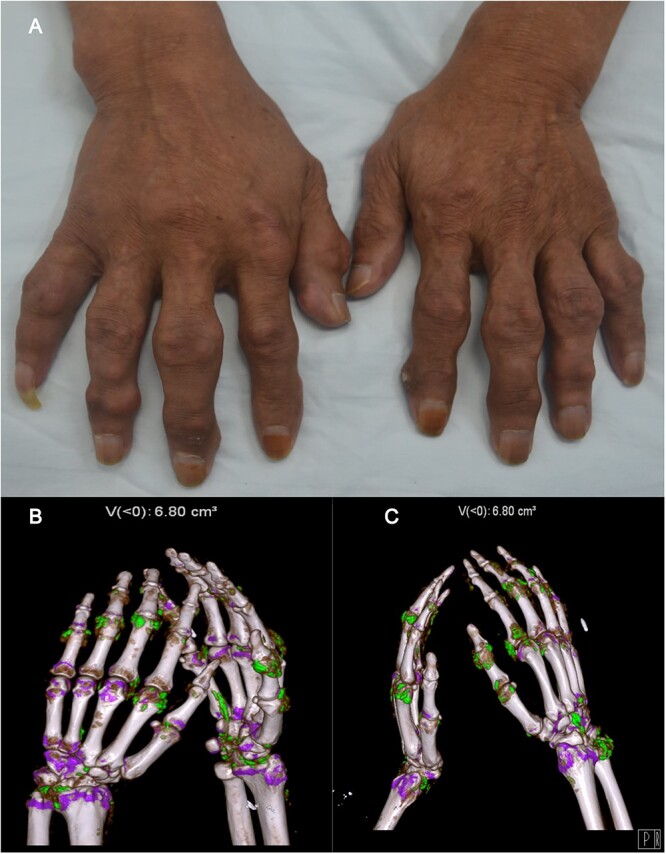
Multiple massive and deforming tophi of the hands (**A**). Dual-energy CT scan of the hands (**B**) demonstrating the extent of urate crystal deposition.

On admission, his blood pressure was 180/108 mmHg without signs of Cushing’s or lymphadenopathy. There were large tophi affecting the small joints of his hands and multiple and round subcutaneous fat masses over his upper body (Fig. 1). MRI revealed widespread and distinct soft masses (fat density) in the subcutaneous layer of the neck region (Fig. 2). Ultrasonography revealed excessive fat deposition in the mandible and elbows. There were also large tophi with joint destruction affecting the small joints of his hands (Fig. 3). His serum urea was 13.1 mmol/l and serum creatinine was 172 μmol/l. Urine dipstick was negative for blood and protein. The urine analysis was unremarkable with no red blood cells or white blood cells. A full immunology screen was normal or negative. His laboratory profile and trend were shown in Table 1. An ultrasound scan of the kidneys showed 9.8 cm left kidney and 10.1 cm right kidney with increased echogenicity in keeping with medical renal disease. A liver ultrasound scan indicated diffuse hepatic change consistent with alcoholic hepatic fibrosis. Following admission, his blood pressure was brought under control and his serum creatinine stabilized. The patient turned down a kidney biopsy and a fine needle aspiration (FNA) of the fat mass. Without pressure symptoms from the masses, the patient declined a lipectomy. His renal function was unchanged and he was subsequently discharged on losartan, atorvastatin, amlodipine, carvedilol and febuxostat, and advised to abstain from alcohol. Both the fatty masses and renal function had remained unchanged 5 months after discharge.

**Table 1:** Results of laboratory tests

Tests	19/05 (OP)	08/06	10/06	13/06	15/06	Reference range
Hemoglobin	103	105	101	103		133–171 g/l
Creatinine	152	172	142	151	154	62–106 μmol/l
Urea	11.8	13.1	10.0	11.0	11.4	2.76–8.07 mmol/l
Uric acid	343.8	367.3	306.5	294	328	202.3–416.5 μmol/l
CO2	20.6	19.7			24.9	22–29 mmol/l
Urine dipstick for blood	(−)	(−)	(−)			
Urine dipstick for protein	(−)	(−)	(−)			
Urine PCR		0.0634	0.807			<0.20 mg/mg
Urine ACR			0.0065			<0.02 mg/mg
24-h urine protein (mg)		50.74	132.92			<140 mg/24 h
24-h urine albumin (mg)			6.1			<30 mg/24 h
Calcium		2.35				2.15–2.55 mmol/l
total cholesterol		4.03				5.2–6.2 mmol/l
triglycerides		1.55				<1.70 mmol/l
LDL-C		2.80				2.59–4.12 mmol/l
Ferritin		21.5				23.9–336.2 ng/ml
Iron		9.57				5.83–34.5 μmol/l
PTH		47.2				12–88 pg/ml
ANCA		(−)				
Anti-GBM Ab		(−)				
PLA2R-Ab		(−)				
dsDNA Ab		(−)				
ENA/RF/ASO		(−)				
C3		1.13				0.9–1.8 g/l
C4		0.36				0.1–0.4 g/l
IgG		10.78				7–16 g/l
IgM		1.1				0.4–2.3 g/l
IgA		2.24				0.7–4 g/l
TSH		0.535				0.55–4.78 mIU/ml
FT3		2.11				2.3–4.2 pg/ml
FT4		1.09				0.89–1.76 ng/ dl
HbA1c		6.0				4.1–6.0%
OGTT (Glu-0 h)		5.06				4.11–6.05 mmol/l
OGTT (Glu-1 h)		8.47				
OGTT (Glu-2 h)		7.16				4.11–7.8 mmol/l

Abbreviations: OP: outpatient; PCR: protein creatinine ratio; ACR: albumin creatinine ratio; LDL-C: low-density lipoprotein cholesterol; PTH: Parathyroid hormone; ANCA: Anti-neutrophil cytoplasmic antibodies; GBM Ab: glomerular basement membrane antibody; PLA2R-Ab: phospholipase A2 receptor antibody; dsDNA: double-stranded deoxyribonucleic acid; ENA: Extractable Nuclear Antigen; RF: Rheumatoid factor; ASO: Serum Antistreptolysin O; C3: Complement 3; C4: Complement 4; IgG: Immunoglobulin G; IgM: Immunoglobulin M; IgA: Immunoglobulin A; TSH: Thyroid stimulating hormone; FT3: Free triiodothyronine; FT4: Free thyroxine; HbA1c: Hemoglobin A1c; OGTT: oral glucose tolerance test; Glu: glucose.

## DISCUSSION

MD is a rare disorder of fat metabolism. Its etiology is unknown though various pathophysiological mechanisms have been proposed including defect in catecholamine-induced lipolysis and mitochondrial disorders of brown adipose tissue [1, 2]. MD is most commonly seen in the Mediterranean and European populations with a male to female ratio of 15:1. Only sporadic cases have been reported in Asian populations and the condition is uncommon in China. Study reports indicated that 90% of the cases affected were middle-aged men with a heavy drinking history [3, 4]. The disease typically runs a slow course and is associated with a variety of metabolic disorders including diabetes mellitus, hyperlipidemia, hypertension, hypothyroidism, and hyperuricemia. The differential diagnoses include obesity, Cushing’s syndrome, diffuse thyroid enlargement, lipomas, liposarcoma, lymphoma, sialadenitis, neurofibromatosis, drug-induced lipomatosis, angiolipoma, and hibernoma.

Our patient experienced multiple slowly growing fat masses in part of his body against the background of several metabolic disorders including poorly controlled hypertension, hyperlipidemia and hyperuricemia. His age, gender, clinical feature and the background of chronic and heavy alcohol consumption would support a diagnosis of MD. Further laboratory tests, ultrasound and MRI ruled out obesity, diabetes, diffuse thyroid enlargement, lymphoma as differentials. These imaging examinations were also useful to differentiate benign lipomatosis from liposarcoma. Lipectomy or liposuction may be required for symptoms control but recurrence is relatively high after surgery [5]. In our patient, through abstinence from alcohol and control of blood pressure and hyperuricemia, the fat masses and renal function had appeared stabilized over the next 5 months.

Without a renal biopsy, we could only postulate the cause of his renal impairment. The increased echogenicity on renal ultrasonography [6] coupled with a stable serum creatinine following admission and blood pressure control supported the diagnosis of chronic kidney disease (CKD). The normal immunology and urine analysis made the diagnosis of an underlying glomerulonephritis unlikely. We postulate his renal impairment and tophaceous gout were consequences of chronic alcoholism [7] and associated metabolic disorders including poorly controlled hypertension and hyperuricemia [8]. Most of the case reports of MD so far have focused on the fatty masses rather than the metabolic disorders associated with this condition. We believe that many metabolic disorders frequently reported in association with MD including hypertension and hyperuricemia may lead to kidney impairment and other serious medical consequences [9] and therefore must be addressed effectively and sufficiently to avoid such consequences. Whereas abstinence from alcohol may inhibit fat progression, yet there is no guarantee for it [10]. On the other side of the coin, adequate control of hypertension and hyperuricemia has been observed to delay renal failure. In the present case, both fat and renal progression appeared to have been halted following alcohol withdrawal and adequate control of hypertension and hyperuricemia.

## AUTHOR CONTRIBUTIONS

RQT: draft, acquisition and analysis of data; PP: concept, critical review and revision; Both authors approved the final version.

## CONFLICT OF INTEREST STATEMENT

None declared.

## FUNDING

None.

## DATA AVAILABILITY

The data of the manuscript can be made available on request.
